# TRPC1 Inhibits Cell Proliferation/Invasion and Is Predictive of a Better Prognosis of Esophageal Squamous Cell Carcinoma

**DOI:** 10.3389/fonc.2021.627713

**Published:** 2021-03-29

**Authors:** Yun-Zhu Zeng, Yong-Qu Zhang, Jiong-Yu Chen, Li-Ying Zhang, Wen-Liang Gao, Xue-Qiong Lin, Shao-Min Huang, Fan Zhang, Xiao-Long Wei

**Affiliations:** ^1^ Department of Pathology, Cancer Hospital of Shantou University Medical College, Shantou, China; ^2^ Department of Breast-Thyroid-Surgery and Cancer Research Center, Xiang’an Hospital of Xiamen University, Xiamen, China; ^3^ Oncological Research Laboratory, Cancer Hospital of Shantou University Medical College, Shantou, China; ^4^ Clinical Laboratory, Cancer Hospital of Shantou University Medical College, Shantou, China; ^5^ Guangdong Provincial Key Laboratory for Breast Cancer Diagnosis and Treatment, Cancer Hospital of Shantou University Medical College, Shantou, China

**Keywords:** TRPC1, esophageal squamous cell carcinoma, prognosis, cell proliferation, migration and invasion

## Abstract

**Background and Objectives:**

In China, over 90% of esophageal cancer (EC) cases are esophageal squamous cell carcinoma (ESCC). ESCC is a frequently malignant tumor with poor prognosis despite the development of comprehensive therapeutic strategies, for which there is still a lack of effective prognostic factors. Previous studies found that the abnormal expression of TRPC1 is closely related to the proliferation, invasion, metastasis, and differentiation of various tumors. However, the relationship between TRPC1 and ESCC is currently unclear. The present study aimed to clarify the clinical significance of TRPC1 and to preliminarily assess the molecular mechanism by which TRPC1 regulates cell proliferation, migration, and invasion in ESCC.

**Materials and Methods:**

Immunohistochemistry (IHC) was used to determine the expression of TRPC1 and Ki-67 in 165 cases of ESCC. The correlations between TRPC1 expression and clinicopathological characteristics were determined, and both univariate and multivariate analyses were utilized to quantify the impact of TRPC1 expression on patient survival. Cell Counting Kit-8, scratch wound healing, and transwell assays were used to determine the effects of TRPC1 on proliferation, migration, and invasion in ESCC *in vitro*, respectively.

**Results:**

The positive expression rate of TRPC1 showed significantly decreased in ESCC (45.50%) compared with the levels in normal esophageal mucosa (NEM; 80.80%) and high-grade intraepithelial neoplasia (HGIEN; 63.20%) (*P*<0.001). Higher expression rate of TRPC1 was associated with low lymph node metastasis (*P*<0.001), high differentiation (*r_s_* = 0.232, *P*=0.003), and low Ki-67 (*r_s_* = −0.492, *P*<0.001). We further revealed that low expression of TRPC1 was associated with poor prognosis (Disease-free survival, DFS: 95% CI=0.545–0.845, *P*=0.001; Overall survival, OS: 95% CI=0.553–0.891, *P*=0.004). Furthermore, we showed that downregulation of TRPC1 promoted the proliferation, migration, and invasion of human esophageal squamous cell carcinoma cell line EC9706 *in vitro*. In contrast, overexpression of TRPC1 inhibited the proliferation, migration, and invasion of human esophageal squamous cell carcinoma cell line KYSE150 (*P*<0.01), in a manner at least in part mediated through the AKT/p27 pathway.

**Conclusion:**

TRPC1 inhibited the proliferation, migration, and invasion of EC9706 and KYSE150 cells, at least, in part mediated through the AKT/p27 pathway *in vitro*. The downregulation of TRPC1 may be one of the most important molecular events in the malignant progression of ESCC. TRPC1 could be a new candidate tumor suppressor gene and a new prognostic factor of ESCC.

## Introduction

China has the highest morbidity and mortality of esophageal carcinoma (EC) globally, particularly for esophageal squamous cell carcinoma (ESCC). Notably, EC is the main disease among a variety of malignant tumors in nine Chinese provinces, including Sichuan and Guangdong. The north of China along the southern border of the Taihang Mountains is another area with a high incidence of EC. In Guangdong Province, the highest incidence is found on Nanao Island, Chaoshan (1, 2), where EC is the leading cause of death in both men and women. Patients with advanced EC have a poor survival rate (3). ESCC usually develops from low-grade intraepithelial neoplasia (LGIEN) to high-grade intraepithelial neoplasia (HGIEN) and then to infiltrative squamous cell carcinoma. Unfortunately, the carcinogenic mechanism behind EC remains unclear. Moreover, it is still difficult to screen early invasive cancer, especially to differentiate HGIEN from early invasive cancer (4). There is thus a need to explore the factors involved in the emergence and progression of EC and their clinical significance, in order to accurately evaluate the diagnosis and prognosis of patients and treat them appropriately.

Transient receptor potential canonical 1 (TRPC1), a member of the TRPC channel family consists of seven subtypes, TRPC1−TRPC7, is involved in the regulation of intracellular Ca^2+^ concentration and plays an important role in cell proliferation, differentiation, apoptosis, and migration (5, 6). The expression of TRPC1 had been extensively studied in various types of tissue or tumor. The abnormal expression of TRPC1 was found to be involved in tumor growth, migration, invasion, and differentiation (7–12).

However, few studies on the relationship between TRPC1 and EC have been performed. This study was established to investigate the relationships between the expression of TRPC1 protein and clinicopathological features/survival in patients with ESCC. We also analyzed the impact of high or low TRPC1 expression on the proliferation, migration, and invasion of ESCC cells *in vitro*.

## Materials and Methods

### Patients and Samples

A total of 165 patients who were diagnosed with ESCC at Cancer Hospital of Shantou University Medical College from May 2014 to December 2016 were enrolled in this study. All patients were free of distant metastasis and had received surgical treatment for ESCC. There were 130 males and 35 females, ranging in age from 42 to 76 years, with a median of 60. Clinicopathological observations were judged according to the TNM classification for EC of the Union for International Cancer Control and American Joint Committee on Cancer (13). According to infiltration depth, 14 patients exhibited pT1, 16 pT2, 133 pT3, and 2 pT4. Lymph node metastases were found in 96 of the 165 tumors (58.2%). In total, 63 cases of ESCC were classified as high differentiation, 75 medium differentiation, and 27 poor differentiation.

Fifty-two cases of normal esophageal mucosa (NEM) were also enrolled in this study. In order to explain the change of TRPC1 expression level in different stages of esophageal carcinogenesis, 19 cases of HGIEN of esophageal squamous epithelium were enrolled.

The specific methods for collecting samples were as follows (14): i) For NEM, samples were collected from the more than 5 cm away from the edge of the cancer lesion. ii) For ESCC, samples were collected within the cancer lesion. iii) For tissue adjacent to carcinoma (TATC), were collected 2 cm away from the edge of the cancer lesion to the normal esophageal tissue.

Postoperative follow-up data were obtained from all patients with a median follow-up of 34.7 months (range 15–48 months). DFS was defined as the time from the date of definitive surgery to the date of disease recurrence, metastasis, or the date of last follow-up. OS was defined as the time between the date of definitive surgery and the date of death from any cause or the date of the last follow-up.

### Immunohistochemical Staining

All samples were fixed in 10% neutral buffered formalin for 8 to 48 h, followed by dehydration with alcohol and xylene. The dehydrated samples were embedded in paraffin.

Immunohistochemical staining was performed using Envision’s two-step method to assess the expression of TRPC1 protein and Ki-67 protein. Briefly, tissues were cut into 4 μm slices and these slices were heated at 65°C for 1 h. Sections were deparaffinized (xylene), rehydrated (incubation in gradient dilutions of ethanol), and incubated (0.3% H_2_O_2_) for 10 min to block endogenous peroxidases. Then, antigen retrieval was performed (TRPC1 with pH=9.0 EDTA, high-temperature repair; Ki-67 with pH=6.0, 0.01 M citrate buffer, high-pressure repair). After allowing the samples to cool at room temperature, they were washed three times with phosphate-buffered saline (PBS, pH=7.4) for 3 min. All primary antibodies, including TRPC1 (Abcam Corporation, Cambridge, UK; ab110837, dilution 1:150) and Ki-67 (Fuzhou Maixin Biotechnology Development Co., Ltd., China; Kit-0005, ready-to-use), were incubated for 1 h at 37°C, while the ready-to-use immunohistochemistry MaxVision™ secondary antibody (Fuzhou Maixin Biotechnology Development Co., Ltd., China; Kit-5107, Kit-5010) was incubated for 15 min at 37°C. Finally, the sections were stained for 5 min with 3,3′-diaminobenzidine (DAB; Fuzhou Maixin Biotechnology Development Co., Ltd., China; DAB-0031) and counterstained with hematoxylin for 1 min. Muscle tissue was used for positive control of TRPC1, while breast cancer tissue was used for positive control of Ki-67. Negative controls were established using PBS buffer instead of the primary antibody.

### Evaluation of Immunohistochemistry

The staining for TRPC1 and Ki-67 was scored as previously reported (7, 15, 16). Immunohistochemical slides were analyzed by two pathologists in a blinded fashion. When the interpretation results were inconsistent, the slide was reassessed until a consensus was reached. First, the most strongly stained area was selected under a low-power microscope, after which 10 visual fields were observed under a high-power lens (×400) for each piece of section, for which the staining of 100 cells was recorded. TRPC1 protein was mainly expressed in cytoplasm/membrane; “−” represents unstained cells, “+” represents low-intensity staining, while “++” represents high-intensity staining. Cells with no or low-intensity staining were defined as those with low expression. Cells with high-intensity staining were defined as those with high expression. Ki-67 protein was mainly expressed in the nucleus; Ki-67-positive cells were expressed as a percentage of the total esophageal squamous carcinoma cells.

### Cell Culture

Human esophageal squamous cell carcinoma cell lines Eca109 and EC9706 were obtained from The Central Laboratory of Cancer Hospital Affiliated to the Medical College of Shantou University. Human esophageal squamous cell carcinoma cell lines KYSE510 and KYSE150 were obtained from Professor Xu Liyan’s laboratory at the Medical College of Shantou University. Cells were cultured in RPMI-1640 (Gibco, California, USA) supplemented with 10% FBS at 37°C and 5% CO_2_.

### Transfections

For the knockdown of TRPC1, TRPC1 siRNAs (siTRPC1-1, siTRPC1-2), negative control (siNC), TRPC1 shRNAs (shTRPC1-1, shTRPC1-2), and shNC were purchased from Suzhou Genepharma Co., Ltd. (China). The sequences of siRNAs were as follows: siTRPC1-1, forward, 5′-GCGACAAGGGUGACUAUUAdTdT-3′, and reverse, 5′-UAAUAGUCACCCUUGUCGCdTdT-3′; siTRPC1-2, forward, 5′-AUAUUUAGAAGUCCGAAAGCCAAGU-3′, and reverse, 5′-ACUUGGCUUUCGGACUUCUAAAUAU-3′; and scramble siRNA (negative control, NC), forward, 5′-UUCUCCGAACGUGUCACGUTT-3′, and reverse, 5′- ACGUGACACGUUCGGAGAATT-3′. The sequences of shDNA were as follows: shTRPC1-1 (pGPU6/GFP/Neo-TRPC1-1), forward, 5′- GCGACAAGGGTGACTATTA-3′; shTRPC1-2 (pGPU6/GFP/Neo-TRPC1-2), forward, 5′-ATATTTAGAAGTCCGAAAGCCAAGT-3′; and scramble shDNA (pGPU6/GFP/Neo-shNC), forward, 5′-GTTCTCCGAACGTGTCACGT-3′.

For the overexpression of TRPC1, pCDNA3.1-TRPC1 and vector pCDNA3.1 were purchased from Addgene (USA). siRNAs, shDNAs and were transfected into EC9706 cells and pCDNA was transfected into KYSE150 at 70% confluence, using Lipofectamine™ 3000 reagent (Invitrogen, California, USA) for 48 h, in accordance with the manufacturer’s instructions. The stably transfected cell lines were obtained by G418 (400 µg/mL) screening. The efficiency of knockdown or overexpression of TRPC1 was confirmed by western blotting.

### Western Blotting

Western blotting was performed as described in our previous report (17). Primary antibodies against TRPC1 (1:3000; Abcam, San Francisco, CA), AKT (1:1,000; Cell Signaling Technology, Boston, MA), p-AKT (1:1,000; CST), p27 (1:1,000; CST), and β-actin (1:3000; CST) were used.

### Cell Proliferation Assay

Cell Counting Kit-8 (CCK-8; Beyotime Institute of Biotechnology, Haimen, China) assay was used to determine cell proliferation after the knockdown or overexpression of TRPC1. Cells of each treatment group were seeded in 96-well plates at a density of 5×10^3^ cells/well and then incubated for 0 (after cell adherence), 24, 48, and 72 h. Next, 10 μL of reagent was added to each well followed by incubation for 2 h at 37°C and 5% CO_2_. The absorbance was measured at 450 nm using a microplate reader (SpectraMaxM5; Molecular Devices, USA). Each experiment was repeated three times independently.

### Wound Healing Assay

EC9706 and KYSE150 cells transfected with TRPC1 were seeded in six-well plates at a density of 3×10^5^ cells/well. Then, the cells were serum-starved for 24 h and a pipette tip was used to form a linear wound in the confluent monolayer. Wound healing was photographed at 0, 6, and 24 h after wound creation. Experiments were performed in triplicate and three random views of each well were recorded.

### Cell Migration and Invasion Assays

Transwell chamber (Corning, New York, USA) and BioCoat Matrigel Invasion Chamber (Corning, New York, USA) were used for migration and invasion assays *in vitro*. Cells (1×10^5^ cells/well) incubated in RPMI-1640 with 1% bovine serum albumin were added to the upper chamber and RPMI-1640 containing 10% fetal bovine serum as a chemoattractant was added to the lower chamber. After 24 or 32 h, the cells on the upper side of the filter were removed, and the cells that remained adherent to the underside of the membrane were fixed in 95% ethanol and then stained with crystal violet dye. Ten random fields per membrane were photographed using an Axio Lab.A1 microscope (Carl Zeiss, Germany) at ×100 magnification. The mean cell number in 10 fields was calculated to obtain a representative value of the number of transmembrane migrating/invading cells. Three independent experiments were performed for each cell line.

### Statistical Analysis

All data were analyzed with SPSS 19.0 statistical software. The measurement data are expressed as mean ± standard deviation by *t*-test. The counting data are expressed as the rate using *χ^2^* test. The rates were compared using Pearson’s chi-squared test. Spearman’s rank was used for correlation analysis. Survival curves were assessed using the Kaplan–Meier method and compared using the log-rank test. Univariate and multivariate analyses were used to quantify the impact of variables on patient survival. Two-sided *P*<0.05 was considered statistically significant.

## Results

### TRPC1 Decrease in ESCC Is Associated With Lymph Node Metastasis and Differentiation

**Figure 1:**
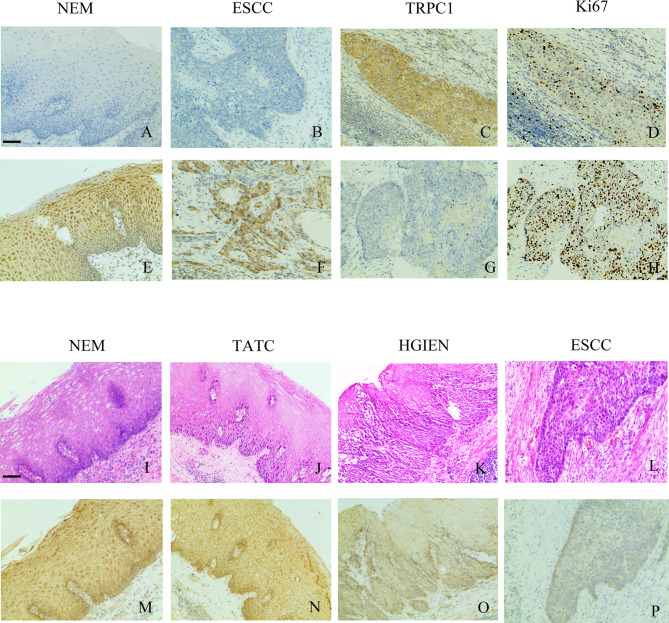
TRPC1 expression and its relationship with Ki-67. **(A)** TRPC1-negative expression in NEM. **(B)** TRPC1-negative expression in ESCC. **(C)** TRPC1 staining in NEM. **(D)** TRPC1 was associated with low Ki-67. **(E)** TRPC1-positive expression in NEM. **(F)** TRPC1-positive expression in ESCC. **(G, H)** TRPC1 staining in ESCC, and tumor without TRPC1 staining showed increased Ki-67. **(I–L)** HE staining was used to observe the histological morphology of NEM **(I)**, TATC **(J)**, HGIEN **(K)**, and ESCC **(L)** in the same patient. **(M–P)** IHC staining showed different expression levels of TRPC1 in the same patient among NEM **(M)**, TATC **(N)**, HGIEN **(O)**, and ESCC **(P)** (magnification: 100×). The scale bar represents 200 μm. NEM, normal esophageal mucosa; TATC, tissue adjacent to carcinoma; HGIEN, high-grade intraepithelial neoplasia; ESCC, esophageal squamous cell carcinoma.

Overall, 52 cases of NEM, 19 cases of HGIEN, and 165 cases of ESCC were detected by immunohistochemistry. The results of immunohistochemical staining are summarized in **Table 1** and **Figures 1A, B, E, F**. Among the 52 cases of NEM, 1 case (1.90%) had no TRPC1 staining (–), 9 cases (17.30%) had low-intensity staining (+), and 42 cases (80.80%) had high-intensity staining (++). Among the 19 cases of HGIEN, 3 cases (15.80%) had no TRPC1 staining (–), 49 cases (21.10%) had low-intensity staining (+), and 12 cases (63.20%) had high-intensity staining (++). Meanwhile, in ESCC, the corresponding numbers were 35 cases (21.20%), 55 cases (33.30%), and 75 cases (45.50%), respectively. TRPC1 showed significantly decreased expression in ESCC compared with the levels in NEM and HGIEN (positive expression rate 45.50% *vs.* 80.80% and 63.20%, respectively; *P*<0.001). These results suggest that TRPC1 is expressed at a low level in ESCC.

**Table 1:** Protein expression of TRPC1 in esophagus tissues.

TRPC1	NEM	HGIEN	ESCC	*X^2^*	*P*
	(n=52)	(n=19)	(n=165)		
**-**	1 (1.90)	3 (15.80)	35 (21.20)		
**+**	9 (17.30)	4 (21.10)	55 (33.30)	22.159	**<0.001**
**++**	42 (80.80)	12 (63.20)	75 (45.50)		

NEM, Normal esophageal mucosa; HGIEN, high-grade intraepithelial neoplasia; ESCC, esophageal squamous cell carcinoma.

Statistically significant (P < 0.05) values are in bold.

In addition, the findings demonstrated that TRPC1 expression was associated with lymph node metastasis (*P*<0.001) and differentiation (*P*=0.028) in this cohort. The expression of TRPC1 in the group with lymph node involvement was significantly lower than that in the group without lymph node metastasis. The expression of TRPC1 in the poor-differentiation group was significantly lower than that in the medium- or high-differentiation group. There was a significant positive correlation between the expression of TRPC1 and differentiation (*r_s_* = 0.232, *P=*0.003; **Table 2**). However, there were no statistically significant differences between the expression of TRPC1 in terms of sex, age, tumor size, and perineural invasion status (*P*>0.05). Nuclear proliferating antigen (Ki-67) is overexpressed in a variety of malignant tumors, which is considered as an indicator of tumor invasion and prognosis, and is highly correlated with distant metastasis and prognosis. It is also expressed in benign tumors, but its expression level is extremely low (18). We found that Ki-67 was highly expressed in the group with low expression of TRPC1, while it showed low expression in the group with high TRPC1 expression (**Figures 1C, D, G, H**). Our results showed that the percentage of Ki-67-positive cells in the group with low TRPC1 expression was significantly higher than that in the group with high TRPC1 expression, and there was a significant negative correlation between them (*r_s_* = −0.492, *P*<0.001).

**Table 2:** Correlation of TRPC1 protein expression with clinicopathological characteristics in 165 ESCC patients.

Clinical characteristics	TRPC1 (%)						X^2^ or rs	*P*
	-		+		++			
**Sex**								
Male	25	(19.20)	46	(35.40)	59	(45.40)	1.909	0.385
Female	10	(28.60)	9	(25.70)	16	(45.70)		
**Age(y)**								
<60	15	(18.50)	25	(30.90)	41	(50.60)	1.768	0.413
>=60	20	(23.80)	30	(35.70)	34	(40.50)		
**T stage**								
<=T2	4	(13.30)	7	(23.30)	19	(63.30)	4.752	0.093
>T2	31	(23.00)	48	(35.60)	56	(41.50)		
**Lymph node metastasis**								
No	16	(23.20)	11	(15.90)	42	(60.90)	17.179	**<0.001**
Yes	19	(19.80)	44	(45.80)	33	(34.40)		
**Perineural invasion**								
No	30	(20.80)	46	(31.90)	68	(47.20)	1.509	0.470
Yes	5	(23.80)	9	(42.90)	7	(33.30)		
**Differentiation**								
Poor	9	(33.30)	10	(37.00)	8	(29.60)	10.852	**0.028**
Medium	16	(21.30)	30	(40.00)	29	(38.70)	0.232*	0.003*
High	10	(15.90)	15	(23.80)	38	(60.30)		
**Ki-67,%,Mean(SD)**	67.43	(17.67)	61.18	(17.43)	50.93	(20.30)	-0.492*	<0.001*

ESCC, esophageal squamous cell carcinoma.

*spearman test, Statistically significant (P < 0.05) values are in bold.

One of the enrolled patients had two separate tumors in the esophageal epithelium, one with a pathological diagnosis of HGIEN and the other with ESCC. We performed TRPC1 immunohistochemical detection on the NEM, TATC, HGIEN, and ESCC tissues of this patient. As shown in **Figures 1I–P**, we found no difference in the expression of TRPC1 between NEM and TATC. In HGIEN, the staining of TRPC1 was weaker than that in NEM and TATC. In ESCC, the expression of TRPC1 was weakest. Taken together, these results suggest that TRPC1 may play an important role in the emergence and progression of ESCC.

### Low Expression of TRPC1 Predicts Poor Prognosis in ESCC

**Figure 2:**
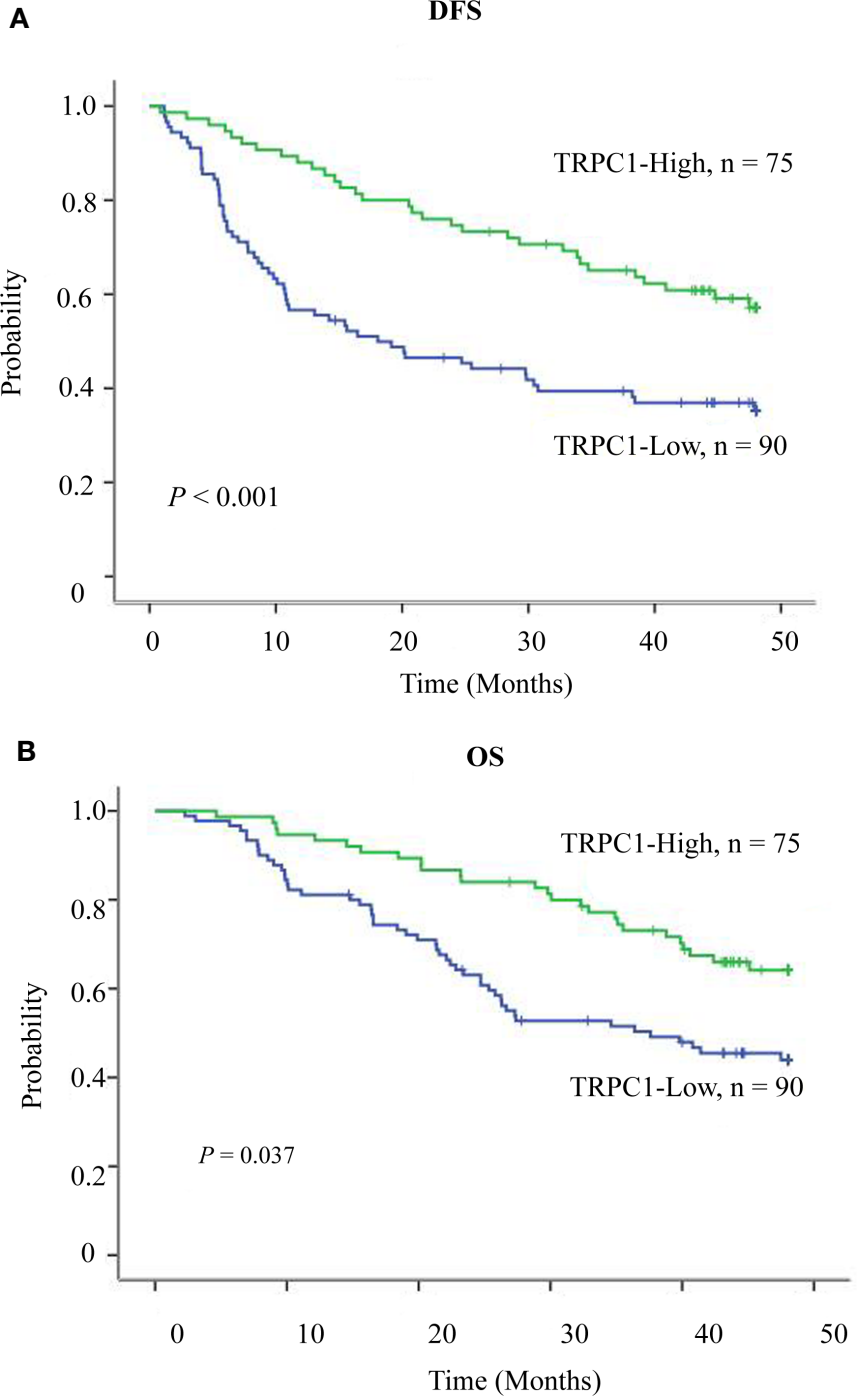
Survival curve of TRPC1 expression in esophageal squamous cell carcinoma patients. **(A)** Overexpression of TRPC1 predicts better disease-free survival in patients with ESCC. **(B)** Overexpression of TRPC1 predicts better overall survival in patients with ESCC.

To further explore the role of TRPC1 in the development and progression of ESCC, we used Kaplan-Meier (log-rank test), univariate, and multivariate regression analyses to assess the survival of the patients with ESCC. The follow-up time of all patients ranged from 15 to 48 months, with a median duration of 34.7 months. Out of 165 ESCC patients, 88 developed disease recurrence or metastasis, while 75 patients died during follow-up; the remaining 90 patients were successfully followed up until the time of writing. Factors including sex, age, T stage, lymph node metastasis, perineural invasion, differentiation, and the pattern of TRPC1 expression were subjected to univariate and multivariate analyses for DFS and OS. The results of survival analysis are presented in **Tables 3**, **4**, **Figure 2**. Univariate analysis showed that particular status of three variables (lymph node metastasis positivity, poor differentiation, and low TRPC1) were associated with a worse prognosis. DFS and OS were significantly worse in the group with low TRPC1 expression than in the group with its high expression (DFS: 95% CI=0.545–0.845, *P*=0.001; OS: 95% CI=0.553–0.891, *P*=0.004). Sex, age, and T stage were not correlated with DFS or OS, while perineural invasion was correlated with OS but not with DFS. DFS was significantly worse in TRPC1-low patients than in TRPC1-high patients (*P<*0.001, **Figure 2A**). Similarly, OS was significantly worse in TRPC1-low patients than in TRPC1-high ones (*P*=0.037, **Figure 2B**). Notably, the multivariate DFS and OS analyses demonstrated that the expression of TRPC1 was an independent prognostic factor for ESCC (DFS: 95% CI = 0.589–0.9181, *P* = 0.007; OS: 95% CI = 0.602–0.978, *P* = 0.033).

**Table 3:** Univariate and multivariate DFS analyses in 165 ESCC patients.

Prognostic factors	Univariate analyses			Multivariate analyses		
	OR	95%CI	*P*	OR	95%CI	*P*
**Sex**						
Male/Female	0.878	0.523-1.474	0.623			
**Age(y)**						
<60/>=60	1.182	0.777-1.799	0.434			
**pT**						
<=T2/>T2	1.817	0.965-3.419	0.064			
**Lymph node metastasis**						
No/Yes	3.046	1.888-4.912	**<0.001**	2.747	1.693-4.456	**<0.001**
**Perineural invasion**						
No/Yes	1.597	0.901-2.830	0.109			
**Differentiation**						
Poor/Medium/High	0.728	0.544-0.973	**0.032**			
**TPRC1**						
Low/High	0.678	0.545-0.845	**0.001**	0.735	0.589-0.918	**0.007**

ESCC, esophageal squamous cell carcinoma.

Statistically significant (P < 0.05) values are in bold.

**Table 4:** Univariate and multivariate OS analyses in 165 ESCC patients.

Prognostic factors	Univariate analyses			Multivariate analyses		
	OR	95%CI	*P*	OR	95%CI	*P*
**Sex**						
Male/Female	0.761	0.426-1.361	0.358			
**Age(y)**						
<60/>=60	1.013	0.644-1.593	0.956			
**pT**						
<=T2/>T2	1.655	0.850-3.220	0.138			
**Lymph node metastasis**						
No/Yes	2.813	1.670-4.741	**<0.001**	2.522	1.483-4.289	**0.001**
**Perineural invasion**						
No/Yes	1.862	1.041-3.334	**0.036**			
**Differentiation**						
Poor/Medium/High	0.727	0.532-0.994	**0.046**			
**TPRC1**						
Low/High	0.702	0.553-0.891	**0.004**	0.768	0.602-0.978	**0.033**

ESCC, esophageal squamous cell carcinoma.

Statistically significant (P < 0.05) values are in bold.

In summary, patients with low expression of TRPC1 appeared to have a greater risk of mortality than those with high expression, and low expression of TRPC1 may be an important predictor of poor prognosis.

### TRPC1 Inhibits Proliferation, Wound Healing, Migration, and Invasion Through Downregulating the AKT Signaling Pathway

**Figure 3:**
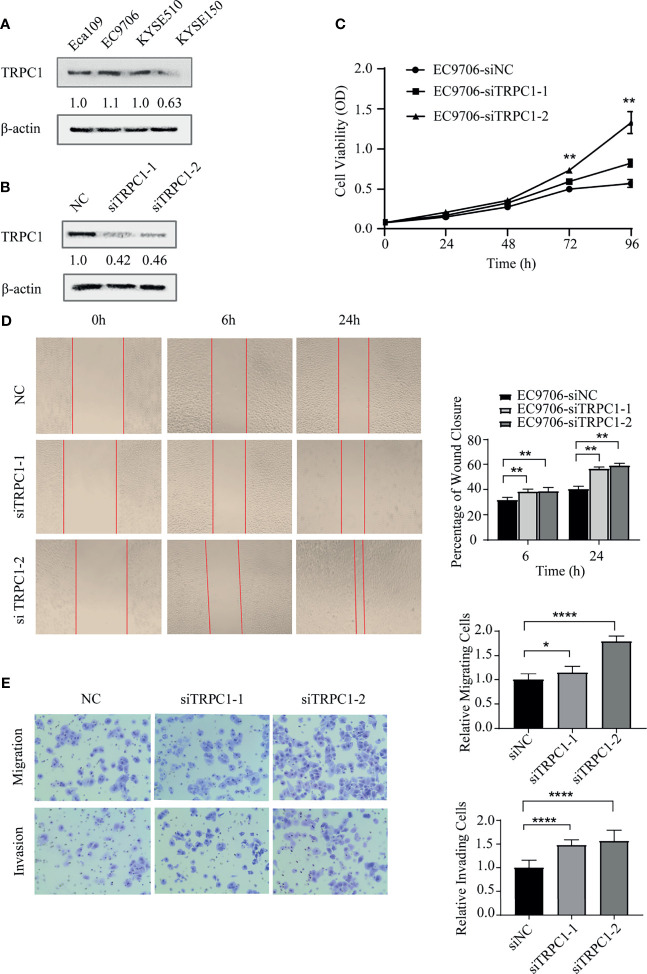
Knockdown of TRPC1 by transfection with siRNA promoted the proliferation, wound healing, migration, and invasion abilities of EC9706 cells. **(A)** Western blotting showed TRPC1 expression in four esophageal squamous carcinoma cell lines: Eca109, EC9706, KYSE510, and KYSE150. **(B)** Representative western blot showing the effect of siRNA directed against TRPC1 on the level of TRPC1 protein in EC9706. **(C)** Analysis of proliferation of cells transfected with siTRPC1 by CCK-8 assay. Cell proliferation was measured 24, 48, 72, and 96h post-transfection. After treatment with siTRPC1, the viability of EC9706 cells was significantly increased. **(D)** Cellular wound healing after knockdown of TRPC1 in EC9706 cells (magnification: 100×). The rate of wound healing of EC9706-siTRPC1-1 or EC9706-siTRPC1-2 cells was significantly higher than that of EC9706-siNC cells (*P*<0.01). **(E)** Cell migration and invasion after knockdown of TRPC1 (magnification: 100×). The cells of the silenced expression group (EC9706-siTRPC1-1 or EC9706-siTRPC1-2) had higher migration and invasion abilities than those of the control group (EC9706-siNC), (*P*<0.05). NC represented as negative control. *P < 0.05, **P < 0.01, ****P < 0.0001.

TRPC1 levels were analyzed by western blotting as shown in **Figure 3A** in the four cell lines: Eca109, EC9706, KYSE510, and KYSE150. We selected EC9706 cells with relatively high expression of TRPC1 for the knockdown experiment and TRPC1 overexpression was detected in KYSE150 cells with relatively low expression. The efficiency of knockdown was confirmed by western blotting (**Figure 3B**). To determine whether TRPC1 affects the viability of EC9706 cells, we evaluated this using CCK-8 assay. The viability of EC9706 cells was significantly increased after transient transfection with siTRPC1-1 or siTRPC1-2 compared with that of scramble siRNA-transfected cells at 24, 48, 72 and 96 h (*P*<0.01, **Figure 3C**). Next, we observed the cell wound healing ability upon knockdown of TRPC1 at 0, 6, and 24 h. We found that the rate of wound healing of EC9706-siTRPC1-1 or EC9706-siTRPC1-2 cells was significantly higher than that of EC9706-siNC cells (*P*<0.01, **Figure 3D**). Finally, we measured the migration and invasion abilities by transwell assay. Interestingly, the relative proportion of migrating cells was increased by 15.3% in siTRPC1-1-transfected and 79.3% in siTRPC1-2-transfected EC9706 cells versus the Negative Control (NC), respectively, compared with the level of siNC-transfected cells (*P*<0.05). The relative proportion of invading cells was distinctly increased by 48.2% in siTRPC1-1-transfected and 56.6% in siTRPC1-2-transfected EC9706 cells compared with the level in EC9706-siNC cells (*P*<0.0001, **Figure 3E**).

**Figure 4:**
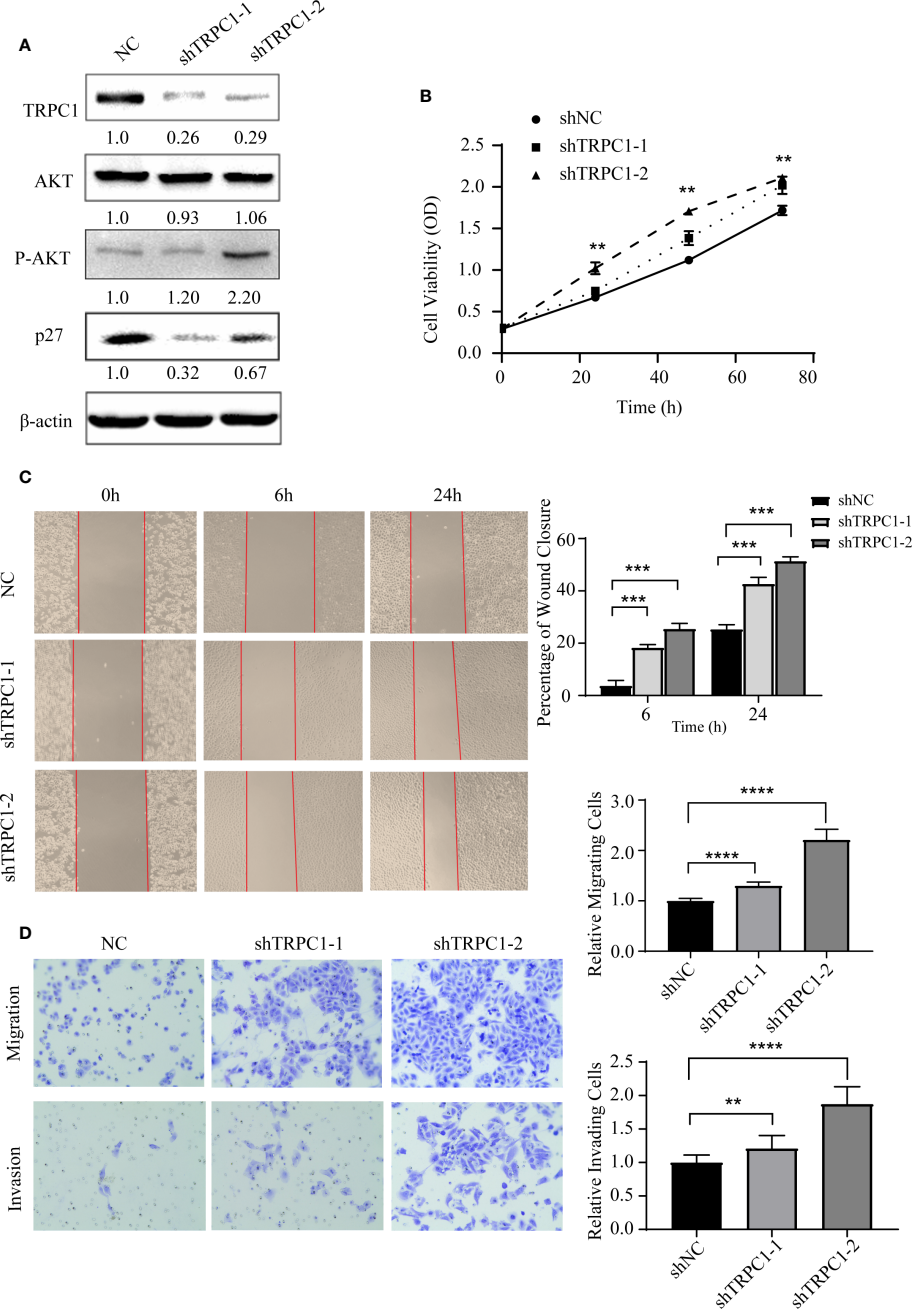
Knockdown of TRPC1 by transfection with shRNA increased the proliferation, wound healing, migration, and invasion abilities of EC9706 cells. **(A)** Representative western blotting showed the effect of shRNA directed against TRPC1 on the level of TRPC1 protein and revealed that knockdown of TRPC1 induced the phosphorylation of AKT (Ser473), which promoted p27 in EC9706, while total AKT remained unchanged. **(B)** Analysis of proliferation of cells transfected with shTRPC1 by CCK-8 assay. Cell proliferation was measured 24, 48, and 72 h post-transfection. After treatment with shTRPC1, the viability of EC9706 cells was significantly increased. **(C)** Wound healing after knockdown of TRPC1 in EC9706 cells (magnification: 100×). The rate of wound healing of the EC9706-shTRPC1-1 or EC9706-shTRPC1-2 cells was significantly higher than that of the EC9706-shNC cells (*P*<0.01). **(D)** Cell migration and invasion after knockdown of TRPC1 (magnification: 100×). The cells of the silenced expression group (EC9706-shTRPC1-1 or EC9706-shTRPC1-2) had higher migration and invasion abilities than those of the control group (EC9706-shNC). NC represented as negative control. **P < 0.01, ***P < 0.001,****P < 0.0001.

Intriguingly, we found that the knockdown of TRPC1 induced the phosphorylation of AKT (Ser473) while total AKT remained unchanged, which increased the level of p27 (**Figure 4A**). The proliferation of EC9706 cells transfected with shTRPC1-1 and shTRPC1-2 was significantly promoted compared with that of cells transfected with shNC (*P*<0.001, **Figure 4B**). In addition, the results of wound healing and transwell assays indicated that the migration and invasion of EC9706 cells transfected with shTRPC1-1 and shTRPC1-2 were also significantly enhanced compared with those of shNC-transfected cells (*P*<0.01, **Figures 4C, D**).

**Figure 5:**
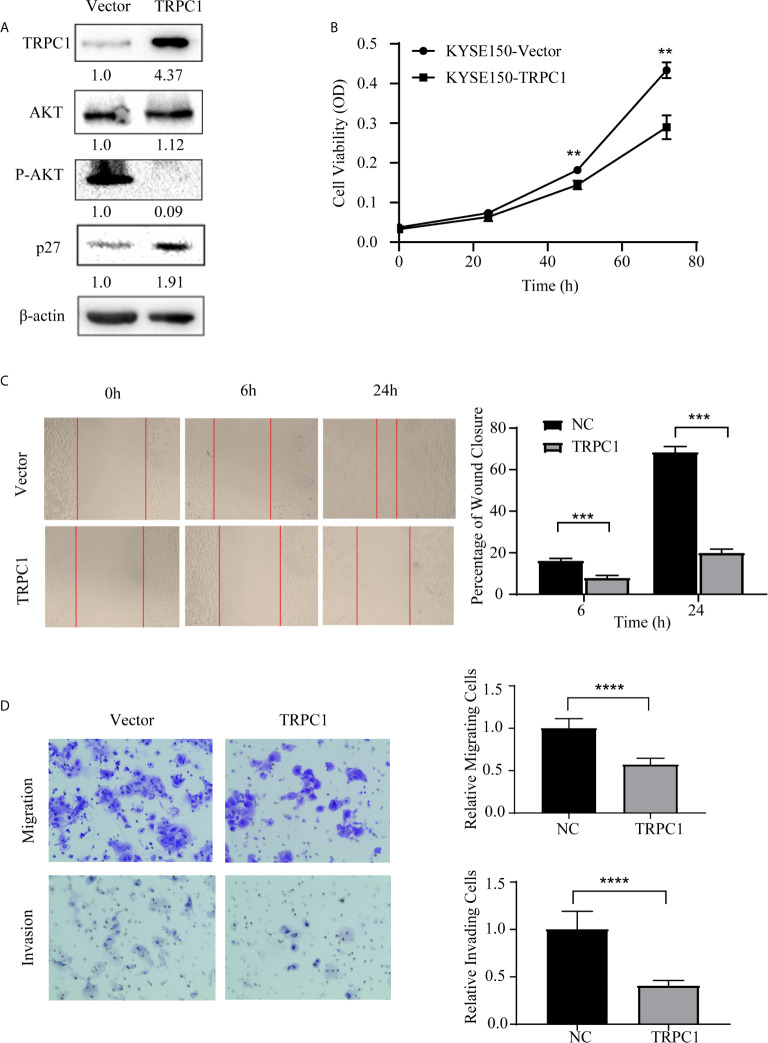
Ectopic overexpression of TRPC1 inhibited the proliferation, wound healing, migration, and invasion abilities of KYSE150 cells. **(A)** Ectopic overexpression of TRPC1 decreased the level of phosphorylated AKT (Ser473) and promoted the expression of p27. **(B)** TRPC1 overexpression inhibited the proliferation of KYSE150 cells, as determined by CCK8 assay. **(C, D)** Overexpression of TRPC1 suppressed cell migration and invasion, as determined by wound healing and transwell assays, in KYSE150 cells (magnification: 100×). NC represented as negative control. **P < 0.01, ***P < 0.001,****P < 0.0001.

Subsequently, we established stable TRPC1-transfected cell lines and measured the transient transfection efficiency by western blotting. We found that the overexpression of TRPC1 inhibited the level of phosphorylated AKT (Ser473) and promoted the expression of p27 (**Figure 5A**). In contrast, the viability of KYSE150 cells transfected with pCDNA3.1-TRPC1 was significantly decreased compared with that of vector-transfected cells after transfection with pCDNA3.1-TRPC1 or pCDNA3.1 (Vector) for 48 and 72 h (**Figure 5B**). In addition, the upregulation of TRPC1 significantly inhibited the wound healing ability (**Figure 5C**), migration, and invasion (**Figure 5D**) of KYSE150 cells compared with those of vector-transfected cells (*P*<0.01). These results implied that the downregulated expression of TRPC1 promoted the proliferation, wound healing, migration, and invasion abilities of EC9706 cells, while TRPC1 overexpression in KYSE150 cells had the opposite effects, which was mediated at least in part through inhibition of the AKT/p27 pathway.

## Discussion

The transient C potential subfamily (TRPC), as one of the main groups of the TRP family, is an important set of nonselective cation channel proteins located on the cell membrane (19). TRPC1, one of the TRPC channels, is expressed in almost all tissues and various tumors. Many studies have demonstrated that the TRPC1 channel plays an important role in tumor cell proliferation, differentiation, apoptosis, migration, and invasion in distinct kinds of tumors, including breast cancer, prostate cancer, gastric cancer, and liver cancer (7–12, 20–22). The abnormal expression of TRPC1 can accelerate the uncontrolled proliferation and invasion of tumor cells.

Our previous work revealed that the expression pattern of TRPC1 may differ between different tumors or in different pathological stages of the same tumor (20). Upon further analyzing the clinical effect of TRPC1 in ESCC, we demonstrated that TRPC1 was expressed at a low level in this disease. The rate of TRPC1 overexpression in ESCC (45.50%) was significantly lower than that in HGIEN (63.20%) and NEM (80.80%). We initially performed TRPC1 immunohistochemical detection on NEM, TATC, HGIEN, and ESCC tissues of the same enrolled patient. TRPC1 was shown not to differ between NEM and TATC. However, the staining of TRPC1 was weaker than that of NEM and TATC in HGIEN.

Beck et al. (10) demonstrated that the growth of human keratinocytes is regulated by calcium. In early differentiated keratinocytes, the storage and influx of endoplasmic reticulum calcium increased, which was similar to the store-operated Ca^2+^ entry (SOCE) manipulated by the calcium pool. The expression of TRPC channels involved in SOCE is necessary for cell differentiation. TRPC1 and TRPC4 proteins play important roles in Ca^2+^ balance and cell differentiation in keratinocytes. The downregulation of TRPC1 and TRPC4 prevents Ca^2+^-induced differentiation. Our results showed that TRPC1 protein expression is significantly related to lymph node metastasis and differentiation, but has no correlation with patient sex, age, T stage, and perineural invasion. The expression of TRPC1 was significantly lower in the group with lymph node metastasis than in the group without lymph node involvement, as well as being lower in the poor-differentiation group than in the medium- or high-differentiation group. Therefore, we speculated that the downregulation of TRPC1 may be related to the emergence and progression of ESCC, as in other cell differentiation disorders. In breast cancer, our previous research also found that TRPC1 inhibited the proliferation, migration, and invasion of cancer cells (20). In a study of the possible effect of TRPC1 on cell proliferation, we simultaneously determined the expression of Ki-67 and analyzed the correlation between these two variables. We found that the rate of Ki-67-positive cells in the group with low TRPC1 expression was significantly higher than in the group with its high expression, with a significant negative correlation between them. Nuclear proliferation antigen (Ki-67) is a nuclear antigen related to apoptosis and cell proliferation, which is found in all phases of the cell cycle except G_0_ (23). In prostate cancer, the expression of TRPC1 was found to be negatively correlated with Ki-67; notably, high expression of TRPC1 prolonged disease-free survival compared with negative TRPC1 expression. TRPC1 may thus have a certain protective effect against the development of prostate cancer cells (7). In the current study, patients with low expression of TRPC1 had a worse ESCC prognosis, in terms of DFS and OS. Moreover, multivariate analysis showed that TRPC1 is an independent prognostic factor for ESCC patients. This result is similar to that in the above study on prostate cancer. However, TRPC1 expression was reported to be upregulated in basal-like breast cancer cells and closely related to epithelial-to-mesenchymal transition (EMT); moreover, high TRPC1 expression was shown to be predictive of poor prognosis (8, 9). The emergence and progression of tumors are the result of the combined effects of multiple factors including multiple genes. Different tumors have different processes of emergence and progression, and the same gene may play different roles in different tumors. In breast cancer, Notch2 plays a tumor suppressive role (24). Overexpression of Notch2 in MDA-MB-231 cells increases apoptosis and inhibits growth and has also been shown to be associated with a better patient prognosis (25). However, in pancreatic cancer, patients with high Notch2 expression have a worse prognosis (26).

Studies of TRPC1 in various tumors suggest that its expression and clinical significance may vary among different tumors. We initially hypothesized that, in the emergence and progression of ESCC, TRPC1 may be related to tumor growth, invasion, and metastasis. The loss of TRPC1 protein is a potential molecular marker for tumor cell proliferation and metastasis, which is expected to be a predictive index for the prognosis of ESCC. We also speculated that TRPC1 may be involved in regulating cell proliferation, migration, and invasion. To confirm this hypothesis, we performed cellular functional experiments. Our results showed that the downregulation of TRPC1 in EC9706 cells accelerated cell proliferation and enhanced migration and invasion by inducing p-AKT and inhibiting p27. However, the upregulation of TRPC1 in KYSE150 cells inhibited cell proliferation and reduced migration and invasion through inhibiting p-AKT and p27 accumulation. The particular mechanisms involved in this require further study. Several studies have confirmed that the inhibition of TRPC1 reduced the influx of Ca^2+^ in cells, which in turn arrested the proliferation of pulmonary artery smooth muscle cells (27, 28). The TRPC1 channel is involved in the influx of storage-dependent Ca^2+^ stimulated by ATP, and subsequently promotes the proliferation of prostate cancer epithelial cells; the depletion of Ca^2+^ storage in the endoplasmic reticulum (ER) is most likely to be the main stress factor that inhibits proliferation (29). In the liver cancer cells Huh7, Ca^2+^ entry into the cells was found to significantly increase and cell proliferation slowed after TRPC1 silencing, which may be related to changes in MAPK signaling pathways (21). El Boustanyet et al. found that the knockdown of TRPC1 protein expression significantly reduced hypoxia-induced invasion in hepatocellular carcinoma (30). In addition, Ge et al. demonstrated that the expression of TRPC1/3/6, vimentin, and α-SMA increased, but E-cadherin decreased, while the Ras, Raf1, and ERK1/2 signaling pathways were activated in gastric cancer cells SGC-7901 stimulated with TGF-β1. As an explanation for this, inhibition of the TRPC1/3/6 signaling pathway may inhibit the EMT process, which may be attributable to post-transcriptional modification (12). Previous studies (9) suggested that AKT pathways may be regulated by TRPC1 and our previous work revealed that TRPC1 plays an essential role in the inhibition of breast cancer progression by inhibiting the PI3K/AKT signaling pathway. In conclusion, these findings show that there may be inconsistencies in the role that the TRPC1 channel plays in various tumors. Summarizing this study, the expression of TRPC1 was found to be downregulated in ESCC, especially in patients with lymph node metastasis. TRPC1 positively correlated with differentiation and negatively correlated with Ki-67. Low expression of TRPC1 was indicative of a poor prognosis in these patients. Interestingly, from *in vitro* experiments, we observed that TRPC1 inhibited the proliferation, migration, and invasion of ESCC in a manner mediated by AKT/p27. Downregulation of TRPC1 may be one of the most important molecular cascade events in the malignant progression of ESCC. TRPC1 may play a significant role as a tumor suppressor gene, and is expected to become a new prognostic factor to guide the clinical diagnosis and treatment of ESCC. However, more research is needed to further clarify the specific regulatory mechanisms involved, which we are currently performing.

## Data Availability Statement

The raw data supporting the conclusions of this article will be made available by the authors, without undue reservation.

## Ethics Statement

The studies involving human participants were reviewed and approved by The Medical Ethical Committee of the Cancer Hospital of Shantou University Medical College. Written informed consent for participation was not required for this study in accordance with the national legislation and the institutional requirements.

## Author Contributions

Conception and design: X-LW. Administrative support: X-LW. Provision of study materials or patients: X-LW. Experimental and supplementary experimental data acquisition: Y-ZZ, Y-QZ, J-YC, L-YZ, and W-LG. Collection and assembly of data: Y-ZZ and Y-QZ. Data analysis and interpretation: Y-ZZ. All authors contributed to the article and approved the submitted version.

## Funding

This work is partly supported by the funds from Youth Research Fund Project of Cancer Hospital of Shantou University Medical College [No.2020A009], Science and Technology Innovation Strategy Special Fund of Guangdong Province [No. Shanfu Branch 2018-49], Science and Technology Special Fund Project Guangdong Province [No. Shanfu Branch 2019-132], Science and Technology Planning Project of Shantou [grant numbers: [2019]62, [2020]58 (Y-ZZ)], Guangdong Medical Science Research Fund [NO.A2016589 (X-LW)], Science and Technology Planning Project of Shantou [grant numbers: [2019]106 (X-LW)], Science and Technology Special Fund of Guangdong Province of China (190829105556145) and Strategic and Special Fund for Science and Technology Innovation of Guangdong Province of China (180918114960704).

## Conflict of Interest

The authors declare that the research was conducted in the absence of any commercial or financial relationships that could be construed as a potential conflict of interest.
