# Corrigendum: TRPC1 Inhibits Cell Proliferation/Invasion and Is Predictive of a Better Prognosis of Esophageal Squamous Cell Carcinoma

**DOI:** 10.3389/fonc.2021.767805

**Published:** 2021-10-06

**Authors:** Yun-Zhu Zeng, Yong-Qu Zhang, Jiong-Yu Chen, Li-Ying Zhang, Wen-Liang Gao, Xue-Qiong Lin, Shao-Min Huang, Fan Zhang, Xiao-Long Wei

**Affiliations:** ^1^ Department of Pathology, Cancer Hospital of Shantou University Medical College, Shantou, China; ^2^ Department of Breast-Thyroid-Surgery and Cancer Research Center, Xiang’an Hospital of Xiamen University, Xiamen, China; ^3^ Oncological Research Laboratory, Cancer Hospital of Shantou University Medical College, Shantou, China; ^4^ Clinical Laboratory, Cancer Hospital of Shantou University Medical College, Shantou, China; ^5^ Guangdong Provincial Key Laboratory for Breast Cancer Diagnosis and Treatment, Cancer Hospital of Shantou University Medical College, Shantou, China

**Keywords:** TRPC1, esophageal squamous cell carcinoma, prognosis, cell proliferation, migration and invasion

In the original article, there was a mistake in **Figure 3D** as published. **The picture of Figure 3D was misused and needs to be corrected.** The corrected **Figure 3D** appears below.

**Figure 3:**
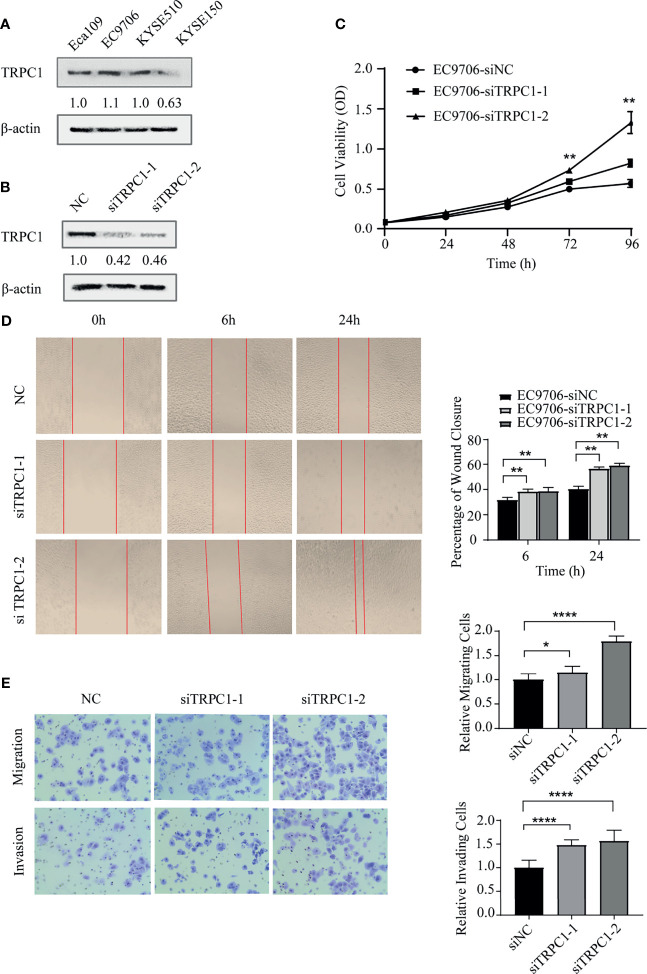
Knockdown of TRPC1 by transfection with siRNA promoted the proliferation, wound healing, migration, and invasion abilities of EC9706 cells. **(A)** Western blotting showed TRPC1 expression in four esophageal squamous carcinoma cell lines: Eca109, EC9706, KYSE510, and KYSE150. **(B)** Representative western blot showing the effect of siRNA directed against TRPC1 on the level of TRPC1 protein in EC9706. **(C)** Analysis of proliferation of cells transfected with siTRPC1 by CCK-8 assay. Cell proliferation was measured 24, 48, 72, and 96h post-transfection. After treatment with siTRPC1, the viability of EC9706 cells was significantly increased. **(D)** Cellular wound healing after knockdown of TRPC1 in EC9706 cells (magnification: 100×). The rate of wound healing of EC9706-siTRPC1-1 or EC9706-siTRPC1-2 cells was significantly higher than that of EC9706-siNC cells (P<0.01). **(E)** Cell migration and invasion after knockdown of TRPC1 (magnification: 100×). The cells of the silenced expression group (EC9706-siTRPC1-1 or EC9706-siTRPC1-2) had higher migration and invasion abilities than those of the control group (EC9706-siNC), (P<0.05). NC represented as negative control. *P < 0.05, **P < 0.01, ****P < 0.0001.

The authors apologize for this error and state that this does not change the scientific conclusions of the article in any way. The original article has been updated.

## Publisher’s Note

All claims expressed in this article are solely those of the authors and do not necessarily represent those of their affiliated organizations, or those of the publisher, the editors and the reviewers. Any product that may be evaluated in this article, or claim that may be made by its manufacturer, is not guaranteed or endorsed by the publisher.

